# Interdigitation Zone Change According to Glaucoma-Stage Advancement

**DOI:** 10.1167/iovs.61.4.20

**Published:** 2020-04-17

**Authors:** Ahnul Ha, Young Kook Kim, Jinho Lee, Eunoo Bak, Young Soo Han, Yong Woo Kim, Jin Wook Jeoung, Ki Ho Park

**Affiliations:** 1 Department of Ophthalmology, Seoul National University College of Medicine, Seoul, Korea; 2 Department of Ophthalmology, Jeju National University Hospital, Jeju-si, Korea; 3 Department of Ophthalmology, Seoul National University Hospital, Seoul, Korea

**Keywords:** glaucoma, interdigitation zone, optical coherence tomography, outer segments, photoreceptors

## Abstract

**Purpose:**

To compare the macular interdigitation zone (IZ) of normal eyes with eyes showing different normal-tension glaucoma (NTG) stages.

**Methods:**

Forty-two normal eyes (age, 56 ± 5.4 years), 45 pre-perimetric eyes (age, 59 ± 6.9 years), 51 mild-to-moderate glaucoma eyes (age, 58 ± 7.2 years; mean deviation [MD], –5.5 ± 3.0 dB), and 50 severe glaucoma eyes (age, 59 ± 6.9 years; MD, –15.1 ± 5.4 dB) were enrolled. All of the subjects underwent high-resolution spectral-domain optical coherence tomography (SD-OCT) to obtain 19 horizontal and 19 vertical macular B-scans 9 mm in length. The en face image of the scan area was divided into 589 rectangular boxes (side length of 375 µm). The IZ locations were marked on the corresponding image boxes. The IZ area was then quantified according to the number of boxes showing IZs among the 589 total boxes.

**Results:**

The IZ area in the severe glaucoma eyes was significantly smaller than in the mild-to-moderate glaucoma eyes (28.99 ± 7.88 mm^2^ vs. 40.79 ± 7.46 mm^2^; *P* < 0.001), was smaller in the mild-to-moderate glaucoma eyes than in the pre-perimetric glaucoma eyes (40.79 ± 7.46 mm^2^ vs. 49.92 ± 8.10 mm^2^; *P* < 0.001), and was smaller still in the pre-perimetric glaucoma eyes than in the normal eyes (49.92 ± 8.10 mm^2^ vs. 56.85 ± 7.94 mm^2^; *P* < 0.001). In the 146 NTG eyes, a statistically significant correlation was found between IZ area and MD (*r* = 0.64; *P* < 0.001).

**Conclusions:**

SD-OCT revealed a reduction in IZ area in NTG eyes, and the extent of the reduction was positively associated with glaucoma severity. These findings suggest, though tentatively, that changes in the outer retinal layer can occur in the course of glaucoma progression.

High-resolution spectral-domain optical coherence tomography (SD-OCT) was employed to examine retinal microstructures in greater detail. SD-OCT indicated that the single, highly reflective, outer retinal band observed using original OCT devices can be resolved as four separate bands that correspond to the external limiting membrane (ELM), the photoreceptor ellipsoid zone (EZ), the interdigitation zone (IZ), and the retinal pigment epithelium (RPE)–Bruch membrane complex.^1^

The third band, the IZ, has been determined to be the covering by apical processes of the RPE of the cone outer segments within a structure known as the contact cylinder.^2^ Although the microscopic IZ structures have yet to be fully elucidated, many studies have found IZ destruction in the following retinal diseases: retinal detachment,^3^ age-related macular degeneration,^4^ foveomacular vitelliform dystrophy,^5^ central serous chorioretinopathy,^6^ and acute posterior multifocal placoid pigment epitheliopathy.^7^ In other words, discontinuity or disruption of the IZ has been reported as a likely hallmark of photoreceptor damage or dysfunction.

Glaucoma manifests in the form of pathological changes in the retinal ganglion cells (RGCs) of the inner retina.^8^^,^^9^ Degenerative changes in the lateral geniculate nucleus and in the visual cortex can both be present in glaucomatous eyes as the result of trans-synaptic degeneration, and their occurrence is considered to be correlated with the severity of RGC loss.^10^ The underlying mechanisms of these processes are still not entirely known, but, based on the fact that retinal neuronal cells are closely related both structurally and functionally, it has been posited that outer retinal layer changes can also occur in glaucomatous optic neuropathy based on similar principles.

Recently, we published findings indicating that in mild-to-moderate and severe glaucoma eyes a relative reduction in EZ intensity occurs and the extent of the reduction is positively associated with glaucoma severity.^11^ This perhaps suggests that, during the progression of glaucoma, the photoreceptor inner segments incur structural and/or functional changes; however, in the IZ, the changes that occur in the photoreceptors over the course of glaucoma remain a mystery. For this reason, the present study was undertaken to expand and improve the current understanding of the IZ features of glaucomatous eyes, including IZ area, IZ area variability, and the correlation of IZ area with advancing glaucoma stages.

## Methods

The Seoul National University Hospital Institutional Review Board approved this cross-sectional study, which adhered faithfully to the tenets of the Declaration of Helsinki. Informed consent was waived due to the retrospective nature of the study.

### Study Subjects

All of the subjects were examined at the Seoul National University Hospital Glaucoma Clinic in Seoul, Korea, between January 2015 and February 2019. Eligible participants were enrolled, consecutively, based on a retrospective medical records review. All underwent a complete ophthalmic examination, which included a best-corrected visual acuity assessment, refraction, slit-lamp biomicroscopy, gonioscopy, Goldmann applanation tonometry (Haag-Streit, Koniz, Switzerland), and dilated stereoscopic optic disc examination. Additionally, the subjects underwent central corneal thickness measurement (Orbscan 73 II; Bausch & Lomb, Rochester, NY, USA), axial length measurement (IOLMaster V.5; Carl Zeiss Meditec, Dublin, CA, USA), stereo disc photography (SDP), red-free retinal nerve fiber layer (RNFL) photography, and optical coherence tomography (Cirrus HD-OCT; Carl Zeiss Meditec) for macular ganglion cell-inner plexiform layer (GCIPL) thickness measurement, as well as central 24-2 threshold testing of the Humphrey visual field (HVF) (HFA II; Carl Zeiss Meditec).

The subjects included in the study were between 40 and 65 years of age, had a best-corrected visual acuity of ≥20/40 (Snellen equivalent), spherical refraction between –6 and 3 diopters (D), untreated intraocular pressure (IOP) of 21 mm Hg or less, an open anterior chamber angle, and reliable visual field (VF) test results. The exclusion criteria were as follows: (1) history of intraocular surgery (except uncomplicated cataract surgery) or retinal laser photocoagulation, or (2) any neurological or systemic diseases potentially affecting the retinal structure and/or function and the VF results. Also excluded were cases of suspicious retinal lesions potentially affecting the outer retinal layer, such as inflammatory conditions or hereditary and degenerative retinal diseases. An experienced ophthalmologist (YSH) reviewed both red-free RNFL photographs and OCT images. Cases with any retinal pathologies including epiretinal membranes, macular holes, macular edema, hard exudate, cotton wool spots, or drusen, as well as changes in the RPE, were excluded. Patients with underlying diabetes but without diabetic retinopathy were not excluded.

Glaucomatous eyes were diagnosed based on the characteristic optic disc appearance (localized or diffuse neuroretinal rim thinning/notching) on SDP and on RNFL defect presence in the corresponding region (based on red-free fundus imaging), irrespective of the presence or absence of glaucomatous VF defect. Cases of optic nerve pits or other congenital anomalous discs were not included. Optic disc signs on SDP and RNFL signs on red-free imaging were evaluated independently by two glaucoma specialists (JL, EB) who were masked to all clinical data. Discrepancies were resolved by consensus. Among the glaucomatous eyes, a diagnosis of pre-perimetric glaucoma was made in cases of normal VFs using conventional HVF analysis. Normal HVF results were defined as follows: mean deviation (MD) and pattern standard deviation within the 95% confidence interval (CI) and glaucoma hemifield test results within normal limits.

Glaucomatous VF defects were defined as (1) a cluster of three points having probabilities of less than 5% in at least one hemifield on the pattern deviation map, including at least one point having a probability of less than 1% or a cluster of two points having a probability of less than 1%; (2) Glaucomatous Hemifield Test results outside the normal limits; and (3) pattern standard deviation beyond 95% of normal limits, as confirmed by no fewer than two reliable examinations (false positives or negatives < 15%; fixation losses < 15%).

For the control group, patients regularly examined due to a family history of glaucoma or cataract were enrolled consecutively. The normal controls showed an IOP ≤ 21 mm Hg, no history of IOP elevation, no glaucomatous optic disc appearance, no RNFL/GCIPL defect, and normal HVF results. If both eyes were found to be eligible, one was randomly selected.

### Imaging of Outer-Retinal Layer

SD-OCT confocal scanning laser ophthalmoscopy scanning (Spectralis HRA+OCT; Heidelberg Engineering, Heidelberg, Germany) was performed in all of the subjects using the eye-tracking feature (TruTrack; Heidelberg Engineering). All of the images were obtained through dilated pupils by one experienced examiner. The 19 horizontal and 19 vertical and consecutive parallel lines of the 9-mm-length macular scans were obtained in the high-resolution setting, and 128 images were averaged. The presence of foveal bulge, foveal depression, or inner-retinal-layer thinning (all as seen on SD-OCT) was considered to represent confirmation of foveal area. For inclusion, all of the images were reviewed to confirm centered scans, a lack of artifacts,^12^^,^^13^ and a signal quality > 20 dB.

### Analysis of Extent of IZ Area

**Figure 1.**
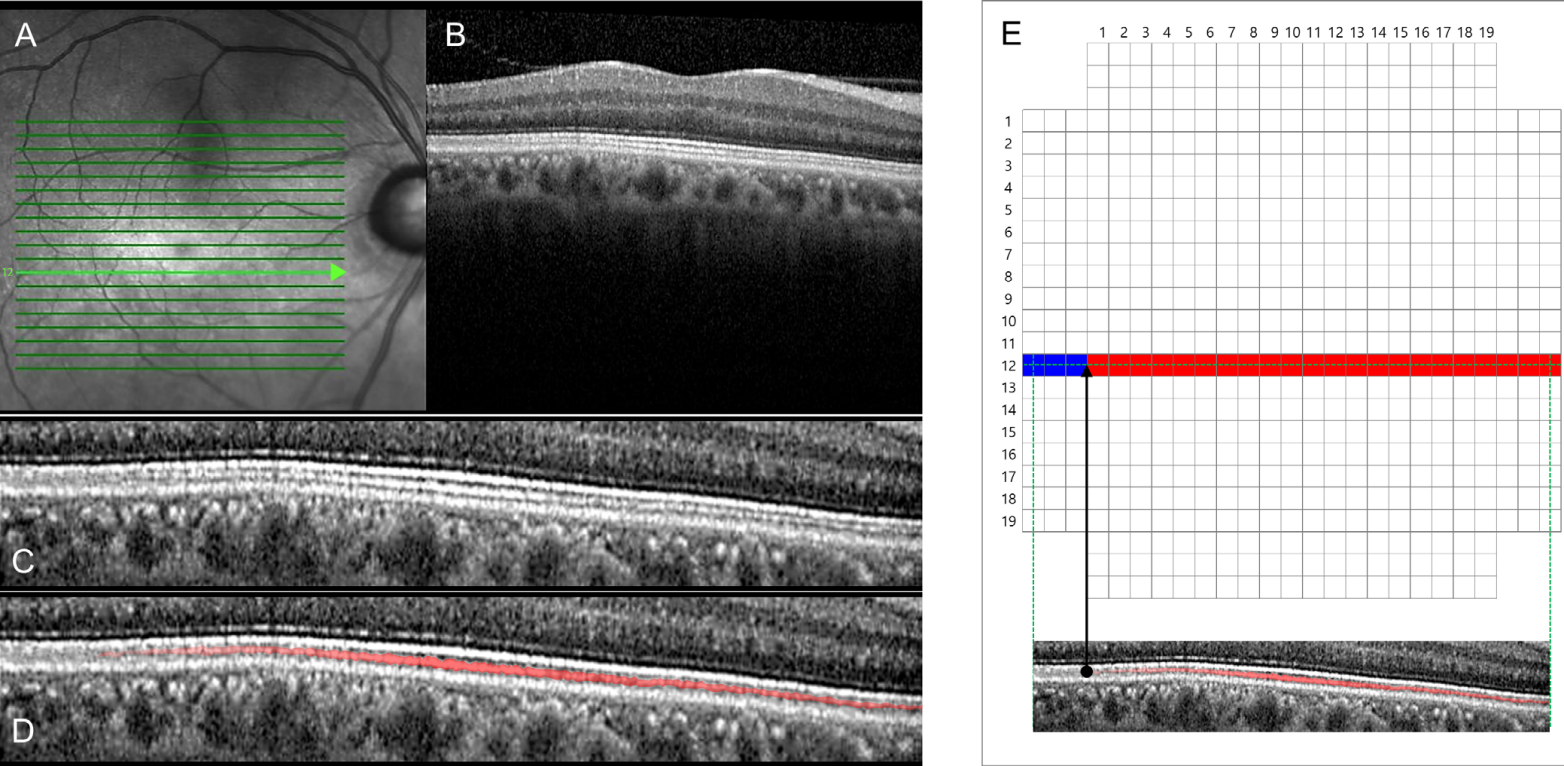
Analysis of IZ area using SD-OCT imaging. (**A**) Horizontal line scan (length, 9 mm) inferior to fovea. (**B**) Original B-scan image. (**C**) Image of outer retinal layer. (**D**) This image is the same as presented in (**C**), but the IZ is depicted as a red solid line. (**E**) The IZ area is shown on a color map, which was divided into 589 rectangular boxes (each side 375 µm in length) containing horizontal and/or vertical scan lines crossing the center. The locations of intact IZs are shown as red boxes; the locations without IZs are shown as blue boxes.

It is known that the IZ becomes less distinct with eccentricity due to the fact that the EZ and IZ approach one another and become indistinguishable at the periphery.^2^ For the purposes of accurate measurement of the IZ, we scrutinized the entire area of the central macular IZ for each eye of each participant using logarithmic-transformed B-scans (19 horizontal and 19 vertical SD-OCT line scans, 9-mm length) displayed as TIFF files. Each en face OCT image of the scan area was divided into 589 rectangular boxes (each side 375 µm in length), each containing horizontal and/or vertical scan lines crossing the center. The grid was superimposed onto OCT images with the fovea as the center point using a commercial image processing tool (Photoshop CS3 10.0.1; Adobe, San Jose, CA, USA), and the extent of intact IZ (the presence or absence of intact IZ in each box location) was confirmed manually. In Figure 1, the intact or absent IZ in each B-scan image is indicated in red or blue, respectively, in the corresponding box.^14^^,^^15^ Only when the entire length of the IZ was intact in a box, both horizontally and vertically, was it recognized as an intact IZ box. IZ discontinuity due to vessel shadowing was not deemed to be IZ loss. An experienced ophthalmologist (AH) who was masked to the patients’ clinical information performed these IZ measurements.

### Quantification of IZ Area

In Figure 1, the area of each box is 0.1406 mm^2^ (i.e., each side is 375 µm), and the analyzed area was assumed to be a two-dimensional flat-plane area; therefore, the total scan area encompassing 589 boxes was 82.828 mm^2^. As an example, if a given eye had 375 red boxes (intact IZs) with 214 blue boxes (absent IZs), the macular IZ area was quantified as 52.73 mm^2^ (= 82.828 mm^2^ × 375/589).

### Cumulative Maps of Intact IZ Frequency for Different Glaucoma Stages

Cumulative maps of intact IZ frequency were visualized for the four groups. Topographic information for all of the left eyes was mirrored horizontally. If all of the subjects of a group showed an intact IZ in a box at a specific location, this box was colored red (255 red, 0 green, 0 blue). Meanwhile, if all of the subjects of a group showed no intact IZ in a box, this box was colored blue (0 red, 0 green, 250 blue). In this manner, the cumulative frequency maps showed red and blue coloring according to the frequency of intact IZs.

### Statistical Analysis

Comparison of normally distributed demographic data and macular IZ area among the four groups was performed by one-way ANOVA with Tukey's post hoc test. The categorical data were analyzed by χ^2^ test using Bonferroni correction for multiple comparisons. For glaucomatous eyes, Pearson correlation analysis was performed on the macular IZ area for VF MD or average GCIPL thickness. In each analysis, parametric or nonparametric tests were employed based on the results of a normality test, and the 95% CI was calculated. The statistical analyses were performed using SPSS Statistics 22.0 (IBM, Armonk, NY, USA); a two-sided *P* value less than 0.05 was considered to represent statistical significance.

## Results

A total of 219 eyes (219 subjects) that met the entry criteria underwent SD-OCT. Thirty-one eyes (31 subjects) were excluded based on poor-quality OCT scans. A total of 42 normal eyes (42 subjects), 45 pre-perimetric glaucomatous eyes (45 subjects), and 101 glaucomatous eyes with VF defect on HVF (101 subjects) were included. Most of the enrolled patients underwent both Spectralis and Cirrus OCT scans for routine screening of ophthalmic diseases in their first visit to our outpatient clinic. A total of eight (4.26%) glaucoma patients who had a Spectralis OCT examination for retinal evaluation but were found to have no retinal pathologies were included. The normal-tension glaucoma (NTG) eyes with VF defect were categorized as follows based on a reliable HVF result that had been obtained within 3 months of SD-OCT imaging: (1) 51 eyes, mild-to-moderate glaucoma (VF MD ≥ –12 dB), and (2) 50 eyes, severe glaucoma (VF MD < –12 dB). See Supplementary Material for demographics of the study subjects.

### Comparison of IZ Area: Normal Subjects and Eyes with Different Glaucoma Stages

**Figure 2.**
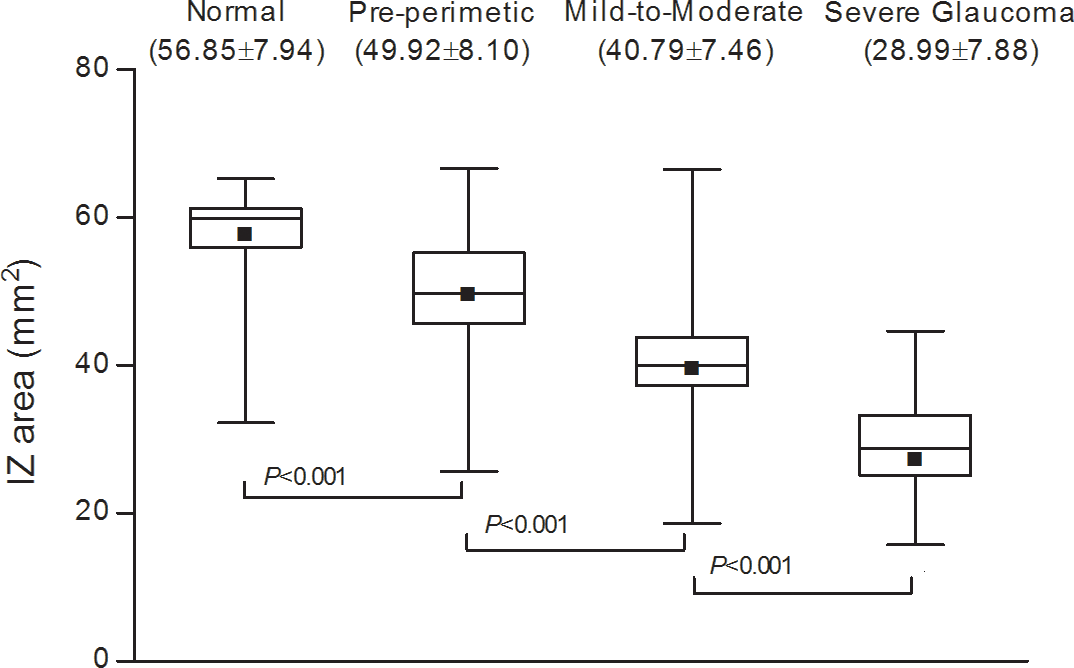
Interdigitation zone areas by box-and-whisker plot for each group. In the pre-perimetric group, the IZ area was significantly reduced relative to the normal group (*P* < 0.001). In the mild-to-moderate and severe glaucoma groups (both *P* < 0.001) relative to the normal subjects, the IZ areas were further reduced. The black squares in the boxes represent means, the lines at the ends of the boxes indicate the upper and lower quartiles, and the two lines outside the boxes mark the maximum and minimum values for each group.

The IZ area differed significantly between the normal and pre-perimetric glaucomatous eyes (56.85 ± 7.94 mm^2^; range, 32.22–65.21; CI, 54.38–59.33 vs. 49.92 ± 8.10 mm^2^; range, 25.64–66.64; CI, 47.49–52.34; *P* < 0.001). In glaucomatous eyes with VF defect, the IZ areas were further reduced for both mild-to-moderate glaucoma (40.79 ± 7.46 mm^2^; range, 18.64–66.49; CI, 38.70–42.90; *P* < 0.001) and severe glaucoma (28.99 ± 7.88 mm^2^; range, 11.98-44.64; CI, 26.76–31.24; *P* < 0.001) groups compared with the normal subjects. In the severe glaucoma group, the IZ area was lower than in the mild-to-moderate glaucoma group (*P* < 0.001) (Fig. 2). See Supplementary Material regarding the reproducibility of measurements.

### Representative Cases

**Figure 3.**
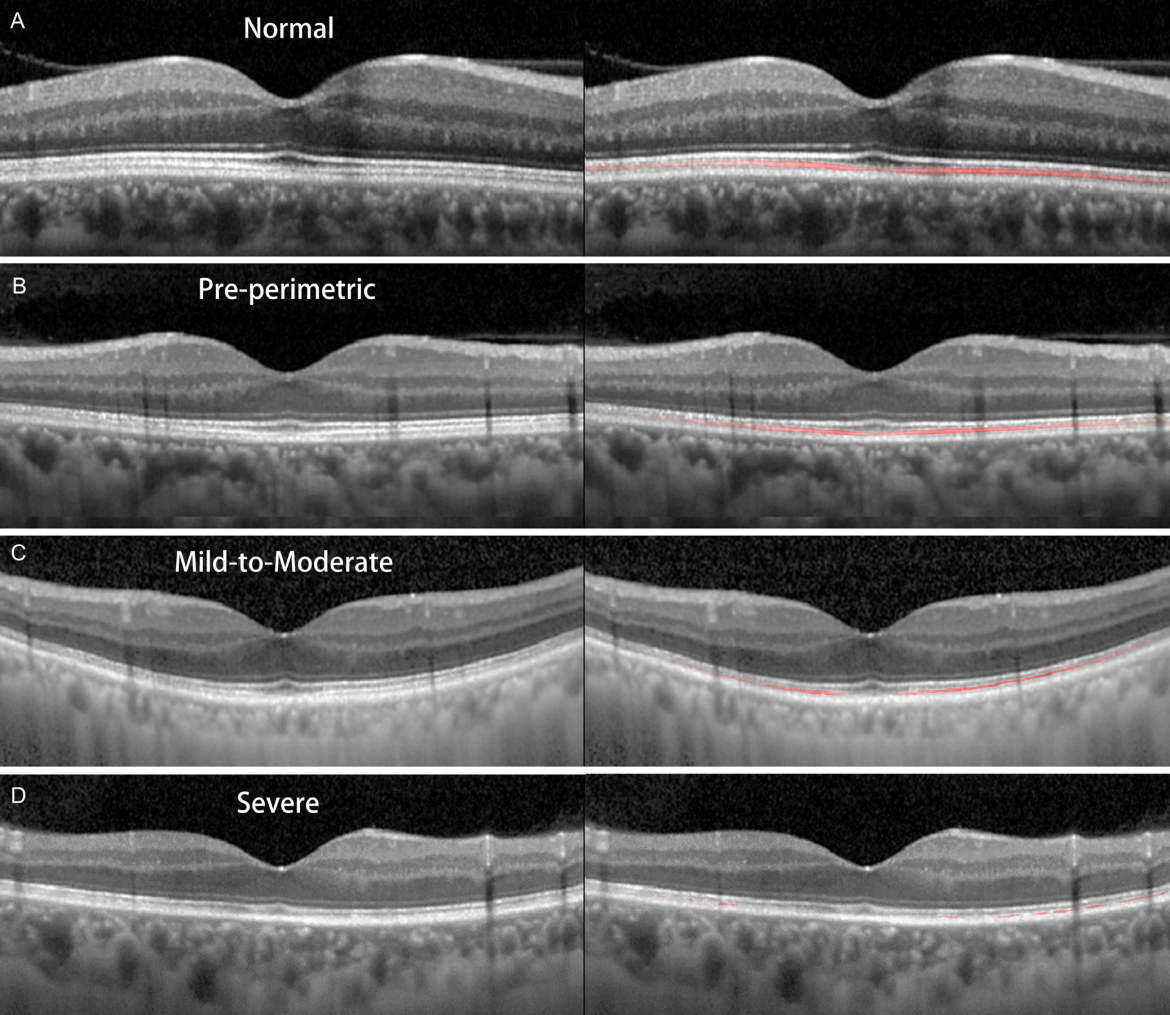
Representative horizontal high-resolution SD-OCT scans through the fovea for each group. (**A**) Normal subject (age 60 years). (**B**) Pre-perimetric glaucoma patient (age 58 years). (**C**) Mild-to-moderate glaucoma patient (age 59 years; VF MD, –6.2 dB). (**D**) Severe glaucoma patient (age 60 years; VF MD, –14.5 dB). The images in the right column are the same as presented in the left column, and the IZ is depicted as a red line. Note that the IZ areas were reduced in proportion to advancement of glaucoma stage.

**Figure 4.**
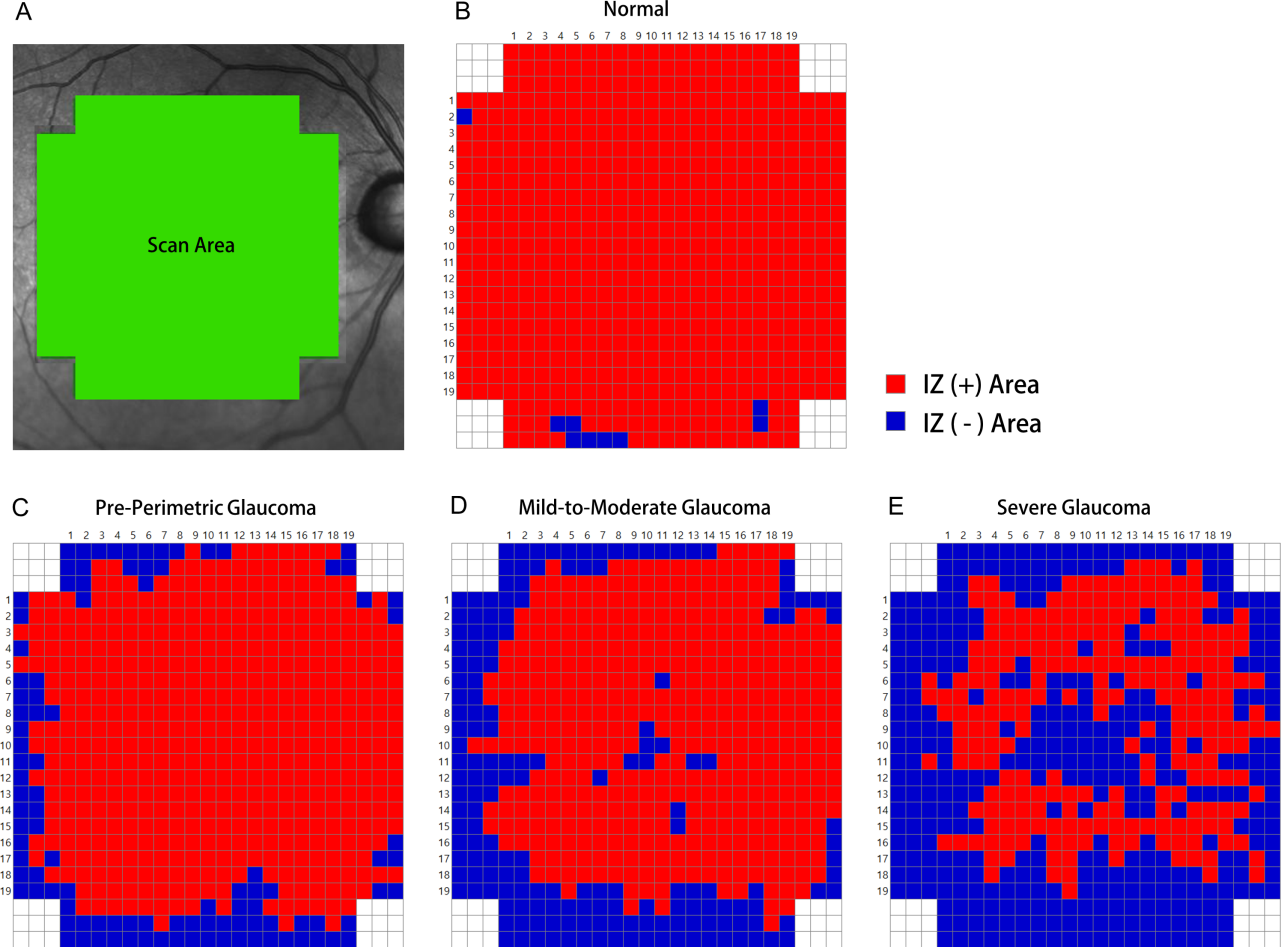
Color map showing IZ areas for representative cases shown in Figure 3. (**A**) Demarcation of scan area. (**B**) Normal subject (age 60 years). (**C**) Pre-perimetric glaucoma patient (age 58 years). (**D**) Mild-to-moderate glaucoma patient (age 59 years; VF MD, –6.2 dB). (**E**) Severe glaucoma patient (age 60 years; VF MD, –14.5 dB). Note that the locations with intact IZs are shown in red, and locations without IZs are shown in blue.

**Figure 5.**
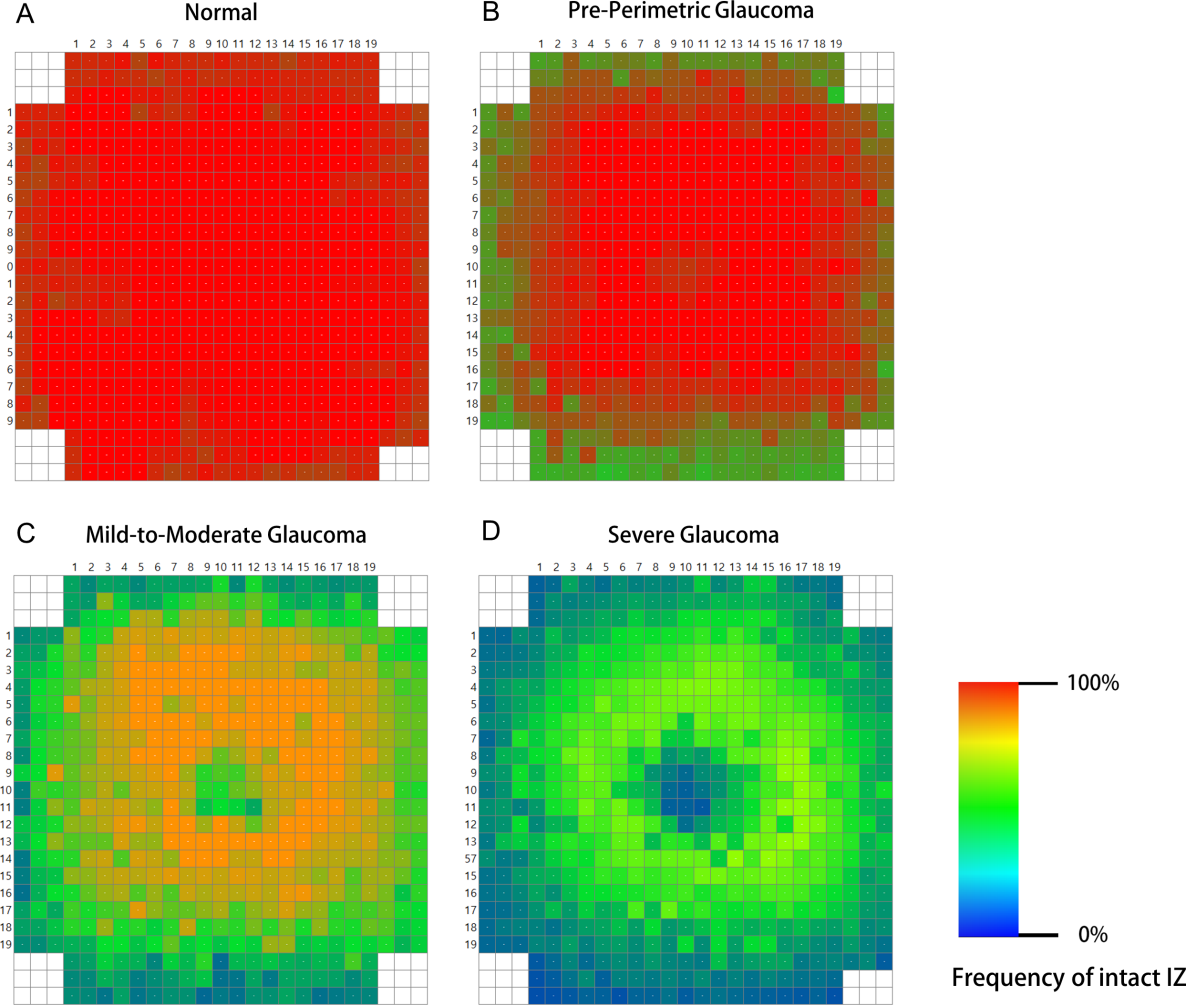
Color map summarizing the cumulative frequency of intact IZs in each location according to glaucoma-stage advancement for all study subjects: (**A**) normal subjects; (**B**) pre-perimetric glaucoma patients; (**C**) mild-to-moderate glaucoma patients; and (**D**) severe glaucoma patients. Cold colors (blue, green) represent relatively lower frequency, and warm colors (yellow, red) represent relatively higher frequency.

Figure 3 provides representative horizontal high-resolution OCT scans through the fovea for the normal and respective glaucoma groups. The first row shows the result for a male normal subject 60 years of age. The second to fourth rows show the results for pre-perimetric glaucoma (age 58 years), mild-to-moderate glaucoma (age 59 years; MD, –6.2 dB), and severe-stage glaucoma (age 60 years, MD –14.5 dB) patients. Figure 4 provides color maps of the entire IZ area for the cases represented in Figure 3. The color maps shown in Figure 5 summarize the cumulative frequency of intact IZ according to glaucoma-stage advancement for each location for all of the study subjects.

### Relationship of IZ Area to VF MD and GCIPL Thickness

**Figure 6.**
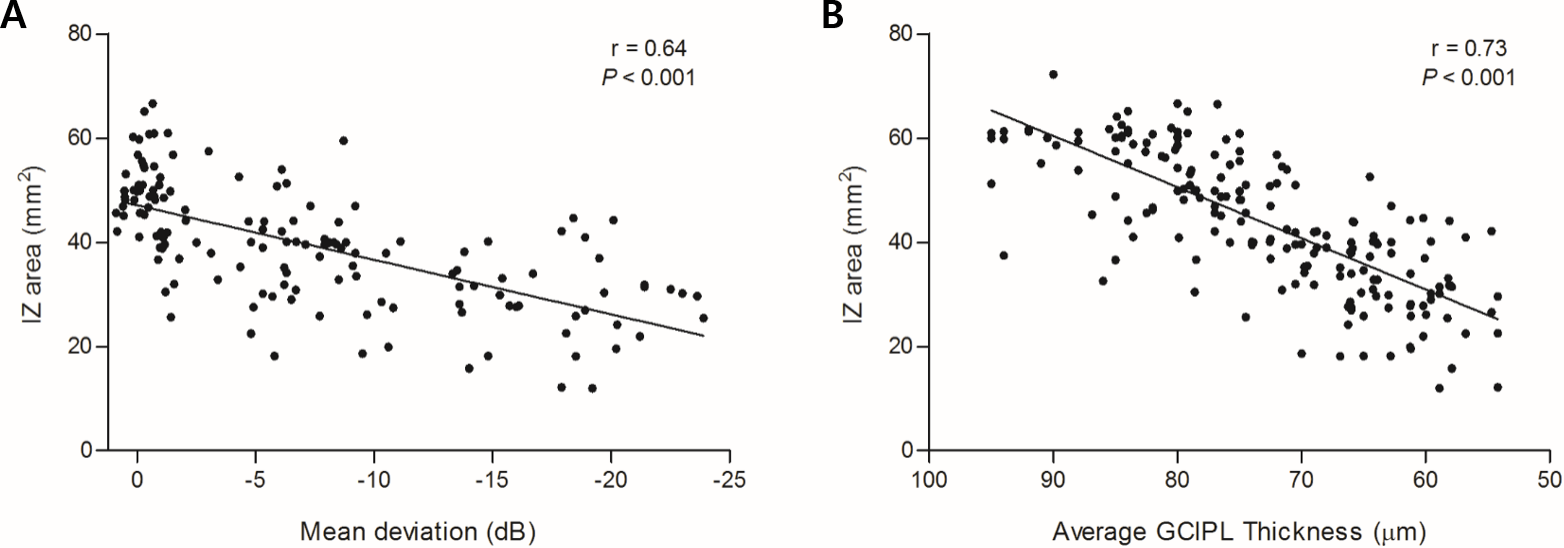
(**A**) For 146 glaucomatous eyes, IZ area was plotted against VF MD. A significant correlation was observed, which remained statistically significant even after controlling for age (*r* = 0.64; *P* < 0.001). (**B**) Scatterplots of IZ area for average macular GCIPL thickness. For all of the subjects, the IZ area and average macular GCIPL thickness were compared based on Cirrus OCT. They showed a significant correlation that remained statistically significant even after controlling for age (*r* = 0.73; *P* < 0.001).

The IZ areas of 146 glaucomatous eyes were plotted against their respective VF MD results (Fig. 6A). A significant correlation was indicated, which remained statistically significant after controlling for age (*r* = 0.64; *P* < 0.001).

The IZ area and average macular GCIPL thickness were compared for all of the subjects (Fig. 6B). A significant correlation was indicated, which remained statistically significant even after controlling for age (*r* = 0.73; *P* < 0.001).

## Discussion

We used SD-OCT to perform in vivo evaluations of the outer retina with regard to the various stages of glaucoma. We observed that a reduction in IZ area occurred in glaucoma and that its extent was positively associated with glaucoma severity. To the best of our knowledge, ours is the first study to have examined IZ change relative to advancing glaucoma stage.

The data gathered in previous studies suggest that the IZ is consistently aligned with the contact cylinder that joins the RPE apical processes to the external portion of the outer cone segment.^2^^,^^16^ Cuenca et al.^17^ showed that IZ structure histologically corresponds to the phagosomes zone that is located in the apical portion of the RPE. They suggested that, during the outer-segment shedding process in photoreceptors, the phagosomes within the RPE are responsible for the third hyper-reflective band (i.e., the IZ) in the outer retina.

Although the microscopic structures of the IZ have not yet been fully determined, IZ integrity has been considered to be an important clinical marker of photoreceptor damage and recovery in various retinal diseases. In fact, IZ restoration is a predictor of visual acuity outcome following surgeries for epiretinal membranes, retinal detachment, diabetic macular edema, or macular hole.^18^^–^^22^ Because the RPE plays an important role in retinal metabolic activity and is critical to visual function, an impaired photoreceptor/RPE unit (presenting as IZ loss) might indicate a pathological change in the photoreceptors. In the present study, we found a significantly negative correlation between IZ area and glaucoma severity, as the IZ area was more reduced in cases of more serious glaucomatous damage. In our results, the patterns of IZ changes tended to begin at the periphery and progress with increasing glaucoma severity. Preferential loss of ganglion cells in the peripheral retina occurs in glaucoma; thus, the pattern of IZ loss found in our study may also reflect that IZ changes in glaucomatous eyes are pathologies accompanying ganglion cell loss. On this basis, we posited that, in the course of glaucoma progression, structural and/or functional changes can occur not only in the RNFL/GCIPL of the inner retina but also in the photoreceptors of the outer retina.

It has been suggested that this reduction in IZ area and its mechanism in glaucoma are related to increased oxidative stress caused by mitochondrial dysfunction. Indeed, there is supporting evidence from both animal and human studies that have assessed reactive oxygen species production, antioxidant levels, and markers of oxidative damage to macromolecules under conditions prevailing in glaucoma.^23^^–^^27^ The photoreceptor/RPE unit is proximal to the choroid (choriocapillaries), whose oxygen partial pressure is high^28^; these cytochemical and anatomical features expose the photoreceptor/RPE unit to a highly oxidative environment. Both photoreceptor and RPE cells show high levels of mitochondrial activity^29^ and, as such, are vulnerable to oxidative stress. Moreover, the phagocytotic function of the RPE imposes an additional oxidative burden on the outer retinal structures. Thus, mitochondrial dysfunction and oxidative damage can be the underlying mechanisms affecting outer retinal changes in glaucomatous eyes.

Another possible cause of the reduction in IZ area is trans-synaptic degeneration in glaucomatous eyes. Neurons can affect, either directly or indirectly, other synapsed neurons by retrograde or anterograde degeneration.^30^ Retrograde trans-synaptic degeneration of either the optic nerve or ganglion cells has been correlated with occipital lesions.^31^^,^^32^ Significant trans-synaptic degeneration of the inner nuclear layer of the retina following optic nerve lesions has also been reported.^33^ Progressive retinal thinning resulting from brain damage due to stroke has been confirmed by SD-OCT ganglion-cell-layer analysis.^34^ Anatomically, RGC dendrites form synapses to the bipolar and amacrine cells, and these interneurons connect, in turn, to photoreceptors.^35^ It has been posited that retrograde trans-synaptic degeneration can take place within the photoreceptors after extensive RGC loss.^36^ Activation of phagocytosis in the outer retina is related to transduction protein shifts within photoreceptors.^37^ Therefore, trans-synaptic degeneration of photoreceptors with RGC loss might represent a further contributory factor to IZ loss over the course of glaucoma progression.

In the current study, we found that a decrease in IZ area occurred not only in perimetric NTG eyes but also in pre-perimetric NTG eyes. Interestingly, in our previous report (with an independent patient group), EZ intensity reduction was observed only in glaucomatous eyes with VF defect, not at the pre-perimetric stage.^11^ The EZ has an anatomical correlation with the inner-segment ellipsoid region of photoreceptor cells.^2^ Given that the ellipsoid is densely packed with mitochondria, the EZ is essential to the structural integrity and function of photoreceptors.^38^^,^^39^ The detection of such IZ changes in pre-perimetric NTG eyes could suggest that, in the early stages, the outer segments of photoreceptors or photoreceptor/RPE units (i.e., the IZ) are more vulnerable to glaucomatous damage than is the inner segment (i.e., the EZ). Otherwise, this observation might be the result of a difference in sensitivity between the quantification method for EZ intensity and that for IZ area; the latter might be more sensitive for detection of changes using SD-OCT, regardless of the actual sequence of histological change. However, we applied different quantitative techniques to evaluate IZ and EZ changes. In a number of diverse retinal diseases, such as retinal detachment, macular holes, or diabetic macular edema, the degree of IZ disruption has been known to be a marker of visual function; therefore, we decided to evaluate IZ area by the presence or absence of intact IZ instead of the intensity. Further, longitudinal studies with a consistent analytical method for elucidating the relationship between IZ and EZ are warranted.

In our opinion, for better monitoring, intervention, and therapy, it is important to understand the alterations associated with glaucomatous optic neuropathy that arise in the outer retinal layer. In the clinical setting, patients showing the same apparent degree of glaucomatous optic nerve damage can manifest different degrees of VF loss.^40^ Notwithstanding that the exact nature of the structure–function relationship in glaucoma is the subject of continuing research, we were able to determine that outer retinal change (i.e., in the IZ area) is a possible objective parameter of the degree of functional impairment in NTG. Furthermore, determining the role of the outer retinal layer in glaucoma pathogenesis could lead to new therapeutic targets in efforts to delay the vision loss associated with further photoreceptor change during the progression of glaucoma.

The findings of this study must be interpreted in light of its limitations. First, although we found excellent inter-reader agreement for IZ intact/absent status, determining IZ margins can be subjective. Also, we calculated the IZ area under the assumption that the analyzed area is a two-dimensional flat plane; however, the analyzed area is actually a curved plane with different curvature in each patient. These factors could have introduced a degree of variability regarding the final results

Second, our results were drawn from patients who turned out to be representative of a relatively narrow age range. Also, given that our patients had NTG, our results might not be directly applicable to primary open-angle glaucoma (POAG) patients with higher baseline IOP. It has been reported that VF defects are deeper and closer to fixation in NTG as compared with glaucoma eyes with high pressure.^41^ In other studies, patients with initial parafoveal scotoma had a significantly lower untreated IOP compared with those with peripheral scotoma.^42^ Therefore, it may be postulated that IOP-independent factors in NTG eyes may preferentially damage the neuroretinal rim or RNFL closer to the papillomacular bundle. Thus, we speculated that, to detect outer retinal changes in macular OCT images, NTG eyes would be more appropriately compared with high-tension POAG; however, further studies exploring outer-retinal changes in high-tension glaucoma patients would be desirable

Third, although we excluded cases with optic nerve pits or other congenital anomalous discs, there is a possibility that optic disc changes due to other causes were included in the pre-perimetric NTG group. In addition, the control group included subjects with a family history of glaucoma. Even though they did not show any signs of glaucoma at the time of their inclusion in our study, they still had an increased risk of developing the disease relative to those without any such family history. Fourth, as also seen in our results in Figure 2, IZ thickness and area have shown variability among healthy adults, suggesting also large individual variability in photoreceptor distribution^14^^,^^15^; as such, serial quantification of IZ area changes might be more useful than single-measurement evaluation. A future longitudinal study investigating the correlation of outer retinal layer changes with the progression of glaucomatous optic neuropathy, therefore, is warranted.

In conclusion, the mean IZ decrease in glaucomatous eyes was determined using SD-OCT imaging, and the decrease was positively associated with glaucoma severity. These findings suggest, tentatively, that outer retinal layer changes might occur in the course of glaucoma progression; they also serve to identify new directions of research that can lead to better understanding of photoreceptor changes during the progression of glaucoma. Further investigation is called for to determine the clinical significance of this reduction in IZ area in glaucoma.

## Supplementary Material

Supplement 1

## References

[1] Staurenghi G, Sadda S, Chakravarthy U, Spaide RF Proposed lexicon for anatomic landmarks in normal posterior segment spectral-domain optical coherence tomography: the IN•OCT consensus. *Ophthalmology*. 2014; 121: 1572–1578. 24755005 10.1016/j.ophtha.2014.02.023

[2] Spaide RF, Curcio CA. Anatomical correlates to the bands seen in the outer retina by optical coherence tomography: literature review and model. *Retina*. 2011; 31: 1609–1619. 21844839 10.1097/IAE.0b013e3182247535 PMC3619110

[3] Lai WW, Leung GY, Chan CW, Yeung IY, Wong D Simultaneous spectral domain OCT and fundus autofluorescence imaging of the macula and microperimetric correspondence after successful repair of rhegmatogenous retinal detachment. *Br J Ophthalmol*. 2010; 94: 311–318. 19822917 10.1136/bjo.2009.163584

[4] Bhutto I, Lutty G Understanding age-related macular degeneration (AMD): relationships between the photoreceptor/retinal pigment epithelium/Bruch's membrane/choriocapillaris complex. *Mol Aspects Med*. 2012; 33: 295–317. 22542780 10.1016/j.mam.2012.04.005 PMC3392421

[5] Puche N, Querques G, Benhamou N, et al. High-resolution spectral domain optical coherence tomography features in adult onset foveomacular vitelliform dystrophy. *Br J Ophthalmol*. 2010; 94: 1190–1196. 20576764 10.1136/bjo.2009.175075

[6] Yang L, Jonas JB, Wei W Optical coherence tomography–assisted enhanced depth imaging of central serous chorioretinopathy. *Invest Ophthalmol Vis Sci*. 2013; 54: 4659–4665. 23737472 10.1167/iovs.12-10991

[7] Cheung CMG, Yeo IY, Koh A Photoreceptor changes in acute and resolved acute posterior multifocal placoid pigment epitheliopathy documented by spectral-domain optical coherence tomography. *Arch Ophthalmol*. 2010; 128: 644–646. 20457992 10.1001/archophthalmol.2010.48

[8] Quigley HA, Dunkelberger GR, Green WR Retinal ganglion cell atrophy correlated with automated perimetry in human eyes with glaucoma. *Am J Ophthalmol*. 1989; 107: 453–464. 2712129 10.1016/0002-9394(89)90488-1

[9] Osborne NN, Wood JP, Chidlow G, Bae JH, Melena J, Nash MS Ganglion cell death in glaucoma: what do we really know? *Br J Ophthalmol*. 1999; 83: 980–986. 10413706 10.1136/bjo.83.8.980 PMC1723166

[10] Yücel YH, Zhang Q, Weinreb RN, Kaufman PL, Gupta N Effects of retinal ganglion cell loss on magno-, parvo-, koniocellular pathways in the lateral geniculate nucleus and visual cortex in glaucoma. *Prog Retin Eye Res*. 2003; 22: 465–481. 12742392 10.1016/s1350-9462(03)00026-0

[11] Ha A, Kim YK, Jeoung JW, Park KH Ellipsoid zone change according to glaucoma stage advancement. *Am J Ophthalmol*. 2018; 192: 1–9. 29750944 10.1016/j.ajo.2018.04.025

[12] Park DW, Lujan BJ Normal interdigitation zone loss by motion-tracked OCT. *Ophthalmol Retina*. 2017; 1: 394. 31047567 10.1016/j.oret.2017.05.005

[13] Rii T, Itoh Y, Inoue M, Hirakata A Foveal cone outer segment tips line and disruption artifacts in spectral-domain optical coherence tomographic images of normal eyes. *Am J Ophthalmol*. 2012; 153: 524–529.e1. 22018706 10.1016/j.ajo.2011.08.021

[14] Shao L, Zhang QL, Zhou LX, Xu L, You QS, Wei WB Using spectral-domain optical coherence tomography to evaluate the type and thickness of interdigitation zone band in adult Chinese. *Sci. Rep*. 2018; 8: 12253. 30115984 10.1038/s41598-018-30848-1 PMC6095864

[15] Gu R, Deng G, Jiang Y, Jiang C, Xu G Area of the cone interdigitation zone in healthy Chinese adults and its correlation with macular volume. *BMC Ophthalmol*. 2018; 18: 188. 30068290 10.1186/s12886-018-0862-7 PMC6090954

[16] Ruskell G Primate retina and choroid. Atlas of fine structure in man and monkey. *J. Anat*. 1992; 180: 215.

[17] Cuenca N, Ortuño-Lizarán I, Pinilla I Cellular characterization of OCT and outer retinal bands using specific immunohistochemistry markers and clinical implications. *Ophthalmology*. 2018; 125: 407–422. 29037595 10.1016/j.ophtha.2017.09.016

[18] Dell'Omo R, Viggiano D, Giorgio D, et al. Restoration of foveal thickness and architecture after macula-off retinal detachment repair. *Invest Ophthalmol Vis Sci*. 2015; 56: 1040–1050. 25613940 10.1167/iovs.14-15633

[19] Terauchi G, Shinoda K, Matsumoto CS, Watanabe E, Matsumoto H, Mizota A Recovery of photoreceptor inner and outer segment layer thickness after reattachment of rhegmatogenous retinal detachment. *Br J Ophthalmol*. 2015; 99: 1323–1327. 25841234 10.1136/bjophthalmol-2014-306252

[20] Shimozono M, Oishi A, Hata M, et al. The significance of cone outer segment tips as a prognostic factor in epiretinal membrane surgery. *Am J Ophthalmol*. 2012; 153: 698–704. 22245463 10.1016/j.ajo.2011.09.011

[21] Serizawa S, Ohkoshi K, Minowa Y, Soejima K Interdigitation zone band restoration after treatment of diabetic macular edema. *Curr Eye Res*. 2016; 41: 1229–1234. 26828673 10.3109/02713683.2015.1113430

[22] Hirota K, Itoh Y, Rii T, Inoue M, Hirakata A Correlation between foveal interdigitation zone band defect and visual acuity after surgery for macular pseudohole. *Retina*. 2015; 35: 908–914. 25549072 10.1097/IAE.0000000000000414

[23] Alvarado J, Murphy C, Polansky J, Juster R Age-related changes in trabecular meshwork cellularity. *Invest Ophthalmol Vis Sci*. 1981; 21: 714–727. 7298275

[24] Moreno MC, Campanelli J, Sande P, Sánez DA, Keller Sarmiento MI, Rosenstein RE Retinal oxidative stress induced by high intraocular pressure. *Free Radic Biol Med*. 2004; 37: 803–812. 15384194 10.1016/j.freeradbiomed.2004.06.001

[25] Ferreira SM, Lerner SF, Brunzini R, Reides CG, Evelson PA, Llesuy SF Time course changes of oxidative stress markers in a rat experimental glaucoma model. *Invest Ophthalmol Vis Sci*. 2010; 51: 4635–4640. 20357192 10.1167/iovs.09-5044

[26] Ko ML, Peng PH, Ma MC, Ritch R, Chen CF Dynamic changes in reactive oxygen species and antioxidant levels in retinas in experimental glaucoma. *Free Radic Biol Med*. 2005; 39: 365–373. 15993335 10.1016/j.freeradbiomed.2005.03.025

[27] Tezel G, Yang X, Cai J Proteomic identification of oxidatively modified retinal proteins in a chronic pressure-induced rat model of glaucoma. *Invest Ophthalmol Vis Sci*. 2005; 46: 3177–3187. 16123417 10.1167/iovs.05-0208 PMC11791686

[28] Nickla DL, Wallman J The multifunctional choroid. *Prog Retin Eye Res*. 2010; 29: 144–168. 20044062 10.1016/j.preteyeres.2009.12.002 PMC2913695

[29] Feher J, Kovacs I, Artico M, Cavallotti C, Papale A, Balacco Gabrieli C Mitochondrial alterations of retinal pigment epithelium in age-related macular degeneration. *Neurobiol Aging*. 2006; 27: 983–993. 15979212 10.1016/j.neurobiolaging.2005.05.012

[30] Jindahra P, Petrie A, Plant GT Retrograde trans-synaptic retinal ganglion cell loss identified by optical coherence tomography. *Brain*. 2009; 132: 628–634. 19224900 10.1093/brain/awp001

[31] Beatty R, Sadun A, Smith L, Vonsattel JP, Richardson EP Jr Direct demonstration of transsynaptic degeneration in the human visual system: a comparison of retrograde and anterograde changes. *J Neurol. Neurosurg Psychiatry*. 1982; 45: 143–146. 7069426 10.1136/jnnp.45.2.143 PMC1083042

[32] Van Buren J Trans-synaptic retrograde degeneration in the visual system of primates. *J Neurol Neurosurg Psychiatry*. 1963; 26: 402. 14066630 10.1136/jnnp.26.5.402 PMC495606

[33] Gills JP Jr, Wadsworth JAC Retrograde transsynaptic degeneration of the inner nuclear layer of the retina. *Invest Ophthalmol Vis Sci*. 1967; 6: 437–448.

[34] Herro AM, Lam BL Retrograde degeneration of retinal ganglion cells in homonymous hemianopsia. *Clin Ophthalmol*. 2015; 9: 1057. 26089638 10.2147/OPTH.S81749 PMC4468984

[35] Masland RH The neuronal organization of the retina. *Neuron*. 2012; 76: 266–280. 23083731 10.1016/j.neuron.2012.10.002 PMC3714606

[36] Nork TM, Ver Hoeve JN, Poulsen GL, et al. Swelling and loss of photoreceptors in chronic human and experimental glaucomas. *Arch Ophthalmol*. 2000; 118: 235–245. 10676789 10.1001/archopht.118.2.235

[37] Yao J, Jia L, Shelby SJ, et al. Circadian and noncircadian modulation of autophagy in photoreceptors and retinal pigment epithelium. *Invest Ophthalmol Vis Sci*. 2014; 55: 3237–3246. 24781939 10.1167/iovs.13-13336 PMC4037936

[38] Jaiswal M, Haelterman NA, Sandoval H, et al. Impaired mitochondrial energy production causes light-induced photoreceptor degeneration independent of oxidative stress. *PLoS Biol*. 2015; 13: e1002197. 26176594 10.1371/journal.pbio.1002197 PMC4503542

[39] Hoang Q, Linsenmeier R, Chung C, Curcio C Photoreceptor inner segments in monkey and human retina: mitochondrial density, optics, and regional variation. *Vis. Neurosci*. 2002; 19: 395–407. 12511073 10.1017/s0952523802194028

[40] Harwerth R, Wheat J, Fredette M, Anderson DR Linking structure and function in glaucoma. *Prog Retin Eye Res*. 2010; 29: 249–271. 20226873 10.1016/j.preteyeres.2010.02.001 PMC2878911

[41] Ahrlich KG, De Moraes CGV, Teng CC, et al. Visual field progression differences between normal-tension and exfoliative high-tension glaucoma. *Invest Ophthalmol Vis Sci*. 2010; 51: 1458–1463. 20042657 10.1167/iovs.09-3806

[42] Park SC, De Moraes CG, Teng CC, Tello C, Liebmann JM, Ritch R Initial parafoveal versus peripheral scotomas in glaucoma: risk factors and visual field characteristics. *Ophthalmology*. 2011; 118: 1782–1789. 21665283 10.1016/j.ophtha.2011.02.013

