# Supplementary material for: Interdigitation Zone Change According to Glaucoma-Stage Advancement
Source: Invest Ophthalmol Vis Sci. 2020 Apr 17;61(4):20. doi: 10.1167/iovs.61.4.20 (PMC7401448; doi:10.1167/iovs.61.4.20)
Supplement: Supplement 1 [file iovs-61-4-20_s001.pdf]

Demographic and Clinical Characteristics of Study Subjects

The Table S1 summarizes the demographic characteristics and systemic and ocular factors for each group. The four groups were similar in terms of mean age, gender distribution, IOP at time of OCT measurement, central corneal thickness, spherical equivalent, axial length, and lens status (P = 0.232, 0.429, 0.099, 0.725, 0.201, 0.378, and 0.871 respectively). Average GCIPL thickness and VF MD were significantly different across the four groups (both $P < 0.001$).

Reproducibility of Measurements

The inter-observer measurement reproducibility was evaluated by independently measuring the IZ area of each OCT image by 2 experienced ophthalmologists (AH and YKK) who had been given only OCT images and who were masked to the clinical information on 30 randomly chosen subjects. The inter-observer ICC for intact-IZ area was 0.91 (95% CIs, 0.84 - 0.95); this represented excellent agreement beyond chance.[1]

Table S1. Demographic and Baseline Clinical Characteristics of Study Patients

	Normal *n* = 42	Glaucoma: Preperimetric *n* = 45	Glaucoma: Mild-to-Moderate *n* = 51	Glaucoma: Severe *n* = 50	*P*
Baseline Factors					
Age (yrs)	56.43 ± 5.42	59.02 ± 6.90	57.86 ± 7.24	58.94 ± 6.87	0.232*
Male, n (%)	23 (55)	28 (62)	33 (65)	25 (50)	0.429†
Systemic Factors					
Diabetes mellitus, n (%)	5 (12)	8 (18)	6 (12)	6 (12)	0.794†
Hypertension, n (%)	7 (17)	9 (20)	9 (18)	8 (16)	0.961†
Cardiovascular disease, n (%)	4 (10)	5 (11)	7 (14)	7 (14)	0.898†
Ocular factors					
IOP (mm Hg)	12.62 ± 2.09	13.34 ± 2.72	12.59 ± 1.91	13.53 ± 2.45	0.099*
Central corneal thickness (μm)	538.28 ± 29.80	541.9 ± 34.04	536.42 ± 35.72	543.11 ± 31.09	0.725*
Spherical equivalent (diopters)	-0.11 ± 1.72	-0.44 ± 2.02	-0.50 ± 1.48	-0.93 ± 2.11	0.201*
Axial length (mm)	23.96 ± 1.37	23.76 ± 1.36	23.85 ± 1.06	24.18 ± 1.28	0.378*
Lens status, pseudophakic, n (%)	10 (23.8)	13 (28.9)	16 (31.4)	15 (30.0)	0.871†
Average GCIPL thickness (μm)	86.11 ± 5.05	78.29 ± 3.16	69.19 ± 3.74	60.96 ± 3.67	< 0.001*
VF MD (dB)	-0.23 ± 0.55	-0.26 ± 0.74	-5.52 ± 3.01	-15.15 ± 5.38	< 0.001*

IOP, intraocular pressure; GCIPL, macular ganglion cell-inner plexiform layer; VF MD, visual field mean deviation.
*One-way analysis of variance, †Chi-square test with Bonferroni correction.

Reference

1. Fleiss JL, Levin B, Paik MC. *Statistical methods for rates and proportions*. 3rd edition. Hoboken, NJ: John Wiley & Sons; 2013.
